# Systems genetics approach reveals cross-talk between bile acids and intestinal microbes

**DOI:** 10.1371/journal.pgen.1008307

**Published:** 2019-08-29

**Authors:** Jingyuan Fu, Folkert Kuipers

**Affiliations:** 1 Department of Genetics, University Medical Center Groningen, University of Groningen, Groningen, the Netherlands; 2 Department of Pediatrics, University Medical Center Groningen, University of Groningen, Groningen, the Netherlands; 3 Department of Laboratory Medicine, University Medical Center Groningen, University of Groningen, Groningen, the Netherlands; UMC Groningen, NETHERLANDS

The emerging role of the gut microbiome in human pathophysiology has led to a paradigm shift in our view of complex traits, moving us from a “genetics–environment” interaction model to one that encompasses “genetics–microbes–environment.” Within this interaction, the microbial mediators are of particular interest. Moreover, the gut microbiome itself is being recognized as a complex trait, and systems genetics has been proposed as a powerful approach to gain understanding of the underlying mechanisms [1]. With respect to the role of microbes in metabolic processes in health and disease, we need to understand what they can do and how genetic determinants of gut microbes influence metabolism or vice versa.

**Fig 1:**
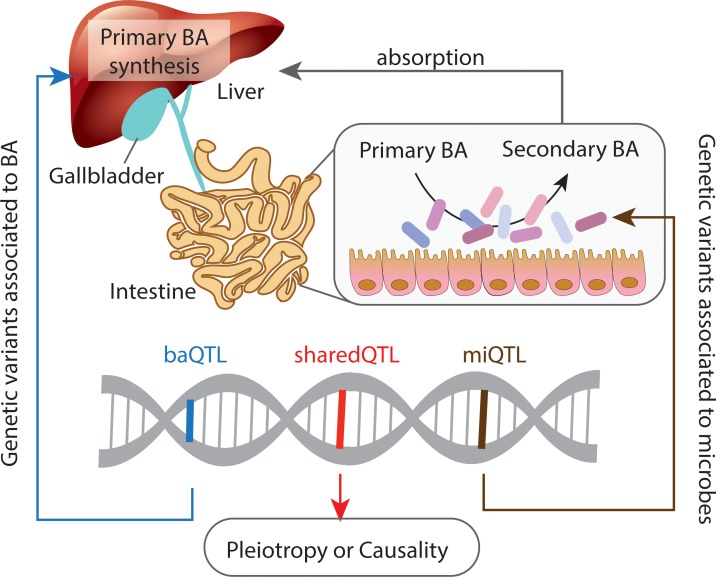
Systems quantitative trait locus (QTL) analysis on gut microbes and BA profiles. baQTL indicates a QTL associated to BA; miQTL indicates a QTL associated to gut microbes; sharedQTL indicates a QTL associated to both BA and gut microbes. BA, bile acid.

A recent proof-of-concept mendelian randomization study highlighted the causality of bacterial production of short-chain fatty acids (SCFAs) in the etiology of type 2 diabetes [2]. SCFAs are products of bacterial fermentation of dietary fibers, affecting the host’s immune fitness and metabolic homeostasis. However, metabolic interactions between host and microbes can be bidirectional and much more complicated. Bile acids (BAs) represent a class of bidirectional metabolic mediators, as their presence in the body is attributable to activities of both host and microbial enzymes (Fig 1). BAs are synthesized in relatively large quantities (approximately 1 g/day in humans) exclusively in the liver through the actions of approximately 20 enzymes encoded by genes that are controlled by distinct metabolic cues that are, in part, derived from the intestine. BAs are then efficiently maintained within the enterohepatic circulation by the actions of several specific transporter systems, i.e., they travel between the liver and the intestinal lumen, where they interact with microbiome. This interaction is 2-fold: bacteria alter BA composition by changing primary (liver-derived) species into secondary, more hydrophobic species, while at the same time, BAs have bacteriostatic actions that depend on their structure and hence, on their physicochemical characteristics [3].

In this issue of *PLOS Genetics*, Kemis and colleagues [4] present a study in which they performed quantitative trait locus (QTL) analyses on the fecal microbiome and plasma as well as cecal BA profiles of a Diversity Outbred (DO) mouse population, a heterogeneous population derived from eight founder strains that individually harbor distinct microbial communities and display different sensitivities to diet-induced metabolic diseases. Kemis and colleague’s study reveals several QTLs associated with variations in bacterial (16S sequencing) and BA profiles, with 17 loci defined as shared QTLs associating with both microbial and BA traits. These shared QTLs highlighted genetic effects on the gut microbes and BA via pleiotropic (QTL → microbe and QTL → BA independently) or causal effects (QTL → BA → Microbe or QTL → Microbe → BA) (Fig 1). The authors then specifically focused on a QTL near the ileal BAs transporter Solute Carrier Family 10 Member 2 (*Slc10a2*) that was associated with both the abundance of *Turicibacter sp*. and the plasma concentration of cholic acid (a primary BA). They conducted both computational analyses and detailed functional studies, and these suggested a complicated, bidirectional causal scenario in which the identified QTL regulates expression of *Slc10a2*, altering BA absorption in the ileum, leading to increased intestinal BA concentrations. These increased BA concentrations, in turn, affect deconjugation of BAs by *Turicibacter sp*., ultimately resulting in a decreased circulating level of cholic acid. This finding is novel, as *Turicibacter* has been reported to be a highly heritable, proinflammatory bacterium [5], but its role in BA metabolism is largely unknown.

This is a comprehensive study to determine genetic determinants of gut microbes and BAs. The authors assessed both plasma and cecal concentrations of 27 BAs, providing an overall picture of genetic and microbial regulation of BA homeostasis. BA metabolism is, however, complex because of the multiple interactions between the various BA species. This is particularly the case in mice compared with humans, because of rodent-specific C6 hydroxylation reactions that generate so-called muricholic acids, hepatic rehydroxylation of secondary BAs to yield primary species upon their return to the liver, and the habit of coprophagy, which allows re-entry of bacteria and (secondary) BA into the murine system. Kemis and colleagues have also chosen to only assess concentrations of individual BA species as complex traits and did not use more comprehensive traits, such as the ratios between conjugated/unconjugated BAs or secondary/primary BAs, even though these ratios may reflect bacterial enzymatic activities relative to genetic background and total BA biosynthesis.

Even with these caveats, this is clearly an important study. While the impact of genetics and of the gut microbiome on BA metabolism is well established, the modes of interaction have largely remained elusive. In addition to their well-known roles in generation of bile flow and absorption of dietary fats and fat-soluble vitamins, BAs are now also known to display hormone-like functions in regulation of glucose, fat, and energy metabolism via activation of specific receptors, including farnesoid X receptor (FXR) and G-protein–coupled BA receptor 1 (GPBAR1) [6]. These BA signaling pathways have now been adopted as novel drug targets for liver and metabolic diseases [7]. Importantly, not all the BA species present in the enterohepatic circulation are equally effective in activating FXR and GPBAR1, with hydrophobic species generally being stronger activators of both receptors [6]. Thus, composition, size, and circulation frequency of the BA pool provide important physiological parameters.

The present study in mice has demonstrated the power of systems genetics to decipher interactions of host genetics, microbiome, and BA metabolism. Yet the differences in genetic background, microbial composition, and BA metabolism between humans and mice also obviously limit clinical translation. On the one hand, mouse models with humanized BA metabolism will be instrumental. On the other hand, similar studies should be conducted in humans, e.g., in well-characterized, relatively large population cohorts and in subjects who may benefit from novel therapeutic strategies for metabolic diseases. Such systems genetics studies in humans will face serious challenges. Several studies have shown that the genetic impact on the gut microbiome is relatively small in humans [8], as environmental factors cannot be controlled and these small genetic effects will limit the power to use genetic variants as instrumental variables for inferring causality in humans. To reveal robust genetic associations to the gut microbiome, large sample sizes will thus be required. To address this, the MiBioGen consortium has been launched to perform genome-wide association study (GWAS) in humans by conducting large-scale meta-analysis on nearly 20,000 individuals from 18 cohorts [9]. Alternatively, recent advances in organ-on-chip technologies, together with the development of in vitro culture of the human gut microbes, opens up new avenues to construct personalized in vitro human systems to mimic the enterohepatic circulation of BAs and simultaneously account for individual genetic makeup and microbial composition. Such a system would be composed of two parts: liver-on-a-chip to assess activity of liver enzymes for primary BA biosynthesis and co-culture of gut microbes on gut-on-a-chip [10] to investigate impact of BAs on the growth rate of microbes and bacterial activity with respect to secondary BA formation. In the near future, we anticipate a toolkit that combines systems genetics analyses in large-scale, well-defined human cohorts with functional studies using individualized in vitro models to understand human host–microbe interactions in health and disease.
